# Variation in reproductive isolation across a species range

**DOI:** 10.1002/ece3.3400

**Published:** 2017-10-07

**Authors:** Karen B. Barnard‐Kubow, Laura F. Galloway

**Affiliations:** ^1^ Department of Biology University of Virginia Charlottesville VA USA

**Keywords:** *Campanula*, cytonuclear incompatibility, genetic distance, reproductive isolation, speciation

## Abstract

Reproductive isolation is often variable within species, a phenomenon that while largely ignored by speciation studies, can be leveraged to gain insight into the potential mechanisms driving the evolution of genetic incompatibilities. We used experimental greenhouse crosses to characterize patterns of reproductive isolation among three divergent genetic lineages of *Campanulastrum americanum* that occur in close geographic proximity in the Appalachian Mountains. Substantial, asymmetrical reproductive isolation for survival due to cytonuclear incompatibility was found among the lineages (up to 94% reduction). Moderate reductions in pollen viability, as well as cytoplasmic male sterility, were also found between some Mountain populations. We then compared these results to previously established patterns of reproductive isolation between these Mountain lineages and a fourth, widespread Western lineage to fully characterize reproductive isolation across the complete geographic and genetic range of *C. americanum*. Reproductive isolation for survival and pollen viability was consistent across studies, indicating the evolution of the underlying genetic incompatibilities is primarily determined by intrinsic factors. In contrast, reproductive isolation for germination was only found when crossing Mountain populations with the Western lineage, suggesting the underlying genetic incompatibility is likely influenced by environmental or demographic differences between the two lineages. Cytoplasmic male sterility was also limited in occurrence, being restricted to a handful of Mountain populations in a narrow geographic range. These findings illustrate the complexity of speciation by demonstrating multiple, independent genetic incompatibilities that lead to a mosaic of genetic divergence and reproductive isolation across a species range.

## 1 INTRODUCTION

A major goal of evolutionary biology was to understand the processes that drive differentiation of populations within a species, generating genetic incompatibility, and ultimately leading to the formation of new species (i.e., speciation) (Coyne & Orr, 2004). Much work has been done characterizing genetic incompatibilities between species, and it is fairly well established that most genetic incompatibilities fit the Bateson‐Dobzhansky‐Muller model (Bateson‐Dobzhansky‐Muller incompatibilities; Dobzhansky, 1937; Muller, 1942; Johnson, 2002), where incompatibilities occur between, rather than within loci (Sweigart & Willis, 2012). Theory suggests these incompatibilities involve alternate divergent alleles that have arisen and become fixed within populations of a species that are isolated and evolving independently from one another. When these isolated lineages come back into contact, incompatibility between these divergent alleles leads to reproductive isolation. If the isolation is strong enough, the lineages will now be different species.

While theory frequently assumes that the divergent alleles contributing to interspecific genetic incompatibility are fixed within species (e.g., Orr, 1995), there is now ample evidence for genetic variation of incompatibility within species (Corbett‐Detig, Zhou, Clark, Hartl, & Ayroles, 2013; Cutter, 2012). Intraspecific variation in reproductive isolation has often historically been ignored in the speciation literature (Scopece, Lexer, Widmer, & Cozzolino, 2010) but has the potential to be a powerful tool for gaining insight into the processes underlying speciation (Cutter, 2012), in particular the early stages when reproductive isolation is evolving within species. Reproductive isolation between species is often the product of multiple, independent genetic incompatibilities (Lowry, Modliszewski, Wright, Wu, & Willis, 2008; Ramsey, Bradshaw, & Schemske, 2003). Even at the earliest stages (i.e., within species), we are likely to find multiple incompatibilities of varying strength (Barnard‐Kubow, So, & Galloway, 2016; Levy, 1991; Skrede, Brochmann, Borgen, & Rieseberg, 2008). Each one of these incompatibilities may be influenced by different evolutionary processes. Therefore, in characterizing the evolution of reproductive isolation, it is important to focus on the individual components and not simply cumulative reproductive isolation.

By characterizing patterns of reproductive isolation, and the factors that predict these patterns, for individual traits within species, we can gain insight into the underlying processes driving the evolution of early arising genetic incompatibilities, which drive the early stages of speciation. For example, if reproductive isolation for a trait is primarily explained by geographic distance between populations, but not genetic distance, evolution of the underlying incompatibility is likely influenced by extrinsic factors, such as divergent environments (Nosil, 2012; Sobel, Chen, Watt, & Schemske, 2010). In contrast, if reproductive isolation for a trait is primarily explained by genetic divergence, independent of geographic distance, then the evolution of the underlying incompatibility is likely primarily driven by factors intrinsic to that species, such as inter‐ or intragenomic conflict or co‐evolution (Barnard‐Kubow et al., 2016; Burton, Pereira, & Barreto, 2013; Crespi & Nosil, 2013; Greiner, Rauwolf, Meurer, & Herrmann, 2011; Hill, 2016). Accordingly, characterizing patterns of intraspecific reproductive isolation across the full geographic and genetic range of a species can give important insights into the evolutionary processes driving the early stages of speciation. However, while multiple studies have documented the existence of variation in reproductive isolation within a species (e.g., Charron, Leducq, & Landry, 2014; Corbett‐Detig et al., 2013; Cutter, 2012; Martin & Willis, 2010; Matute, Gavin‐Smyth, & Liu, 2014; Pinheiro et al., 2013; Snoek et al., 2014), detailed investigations characterizing multiple components of intraspecific reproductive isolation across the full geographic and genetic range of a species are generally lacking (although see Peterson et al., 2013; Martin et al., 2017).

We examine patterns of intraspecific reproductive isolation across the geographic and genetic range of the herbaceous species *C. americanum* to gain insight into the evolutionary processes driving incipient speciation in this system. Previous work has demonstrated substantial intraspecific reproductive isolation in *C. americanum*, primarily in the form of reduced germination and survival when crossing between geographically and genetically distant populations (Barnard‐Kubow et al., 2016; Etterson, Keller, & Galloway, 2007; Galloway & Etterson, 2005). Here, we focus on the role of genetic distance in reproductive isolation while controlling for geography by characterizing patterns of reproductive isolation between divergent genetic lineages that co‐occur within a relatively narrow geographic range. Specifically, we crossed populations from three divergent Mountain lineages and measured reproductive isolation in first generation (F1) hybrids across the life cycle, including germination, survival, and reproductive traits. We then asked the following questions. What traits show reproductive isolation among divergent Mountain lineages? Is reproductive isolation among Mountain lineages predicted by geographic or genetic distance? Finally, by combining the current and previous range‐wide crossing studies, does reproductive isolation vary or remain constant across the geographic and genetic range of *C. americanum*, and how can that inform our understanding of the underlying genetic incompatibilities?

## 2 MATERIALS AND METHODS

### 2.1 Study system

*Campanulastrum americanum* Small (=*Campanula americana* L.) is an autotretraploid, monocarpic herb found in the eastern half of the United States. Individuals are annual or biennial, insect‐pollinated, and highly outcrossing (Galloway, Cirigliano, & Gremski, 2002; Galloway, Etterson, & Hamrick, 2003; Johnson, Delph, & Elderkin, 1995). *Campanulastrum americanum* typically grows in disturbed habitats and seeds are dispersed passively, traits that likely contribute to its patchy population structure. *Campanulastrum americanum* exhibits biparental plastid inheritance. Although inheritance is predominantly maternal, 10–50% of offspring inherit both the maternal and paternal plastids or occasionally just the paternal plastid (Barnard‐Kubow, McCoy, & Galloway, 2017).

**Figure 1:**
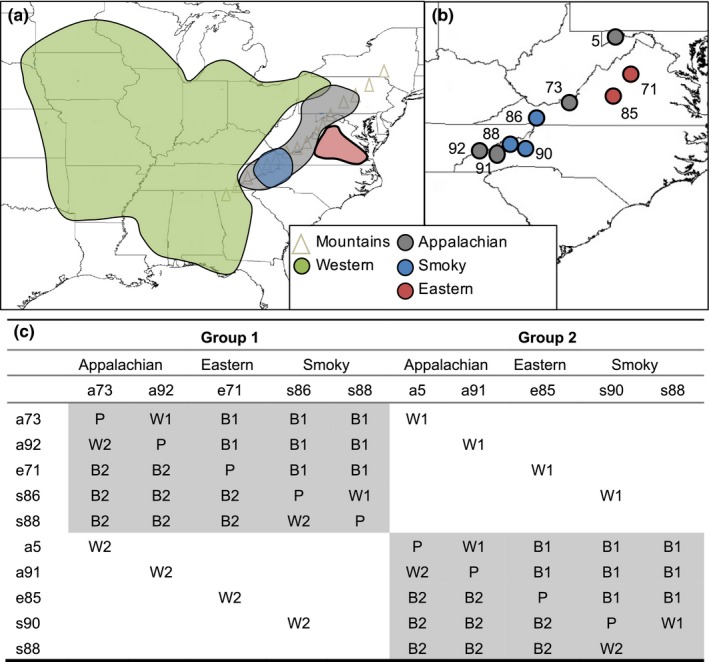
(a) Approximate distribution of each genetic lineage of *C. americanum* in the eastern United States. (b) Location of *C. americanum* populations used for evaluating RI; populations shaded according to genetic clade. (c) Crossing schematic for obtaining parental (P), within‐clade (W), and between‐clade (B) seed, with reciprocal crosses marked as 1 and 2. The shaded boxes correspond to the two main crossing groups

Chloroplast and nuclear markers resolve three genetic clades: Western, Appalachian, and Eastern (Barnard‐Kubow, Debban, & Galloway, 2015). There is also a set of populations in the Smoky Mountains (Smoky) that switch clades between the chloroplast and nuclear phylogenies. As these populations do not fit cleanly into either clade, we treat them as a separate genetic clade. The Appalachian clade is highly divergent, with the remaining clades more closely related. The clades also differ in their geographic extent with the Western clade found throughout much of the species range, and the other three primarily restricted to the Appalachian Mountains (“Mountain” lineages; Figure 1a; Barnard‐Kubow et al., 2015). As a result, the Mountain lineages occupy an environmental niche distinct from that of the Western clade, with the Western clade also occupying a greater range of environments (Barnard‐Kubow et al., 2015).

### 2.2 Evaluation of reproductive isolation

To evaluate patterns of reproductive isolation (RI) among the Mountain lineages, nine populations were identified that were distributed throughout the Mountain region of *C. americanum's* range, with two, three, and four populations sampled from the Eastern, Smoky, and Appalachian clades, respectively (Table S1, Figure 1b). For the crossing design, the nine populations were split into two groups with each group containing two Appalachian populations (“a”), an Eastern population (“e”), and two Smoky populations (“s”) (Figure 1; one Smoky population, s88, was used in both crossing groups). All pairwise crosses were conducted between populations in each group, with additional crosses carried out between groups to increase the number of within‐clade crosses (Figure 1c). In total, eight within‐ and 16 between‐clade crosses were conducted, with reciprocals for each cross. Crosses were also carried out within each of the nine populations to obtain parental seed generated under the same greenhouse conditions. Altogether this crossing design resulted in 57 cross‐types (nine parental crosses + eight within‐clade and 16 between‐clade crosses × 2 crossing directions). Cross‐types are notated with maternal populations first, and lower case letters indicating to which clade the populations belong.

Field‐collected seed from each parental population was germinated and grown under controlled conditions for carrying out within‐ and between‐clade crosses. For each parental population, 30–45 replicates of one seed each were surface sown onto potting medium (3:1 Promix:Turface) in 2.54 cm × 2.54 cm cells in 9x18 germination flats. Replicates were spread evenly among 18–20 families, except for s90 where only five families were available. Seed location was fully randomized, and germination flats were placed in a growth chamber with a 24°C day/14°C night temperature regime and 12‐hr days. Seeds were watered daily. After germination had slowed, flats were moved to 5°C for eight weeks of vernalization to stimulate flowering. Plants were then removed from the cold and a subset of 16 plants from each population was transplanted into containers and moved to a greenhouse where supplemental light increased day length to 16 hr. Individuals to transplant were chosen in a stratified random manner, as they were evenly distributed across maternal families but randomly selected within families. Sixteen genotypes from each population were then used in carrying out within‐ and between‐clade crosses, with reciprocal crosses created using the same set of individuals as pollen donors and pollen recipients in alternate crossing directions. No more than two genotypes came from the same maternal family except for population s90, which included 3–4 genotypes per family. The same 16 genotypes were also crossed pairwise within the populations to produce parental population seed.

Previous studies had demonstrated substantial RI in intraspecific *C. americanum* hybrids in the F1 generation (Barnard‐Kubow et al., 2016; Etterson et al., 2007; Galloway & Etterson, 2005). Therefore, performance of F1 hybrids relative to their parental populations was determined by planting and growing F1 seed under controlled conditions and measuring the following traits: germination, survival, number of flowers, pollen viability, and male sterility. For each cross‐type, 40 replicates of two seeds each were planted, with replicates evenly spread among maternal families. Germination success and seedling mortality were scored for 36 days. Germination was scored when cotyledons had emerged and separated. At times, seedlings were observed where the seed coat was not shed and the cotyledons never emerged. These situations were recorded as mortality following germination events. After germination slowed, all cells were randomly thinned to one seedling, unless cells contained seedlings with chlorotic phenotypes (i.e., white or variegated), where multiple seedlings were left to maximize the likelihood of sufficient seedlings surviving for transplant. After germination had stopped (36 days postplanting), flats were moved to 5°C for eight weeks of vernalization to stimulate flowering. Plants were then removed from the cold and any mortality recorded. A subset of 25 surviving plants from each cross‐type was then transplanted into the greenhouse. Individuals to transplant were randomly chosen, but evenly distributed across maternal families, with one to five individuals per maternal family. Two of 57 cross‐types, s88xa5 and s88xa73, had reduced numbers (22 and 15, respectively), due to high early mortality. In total, 1,412 plants were transplanted. Plants were fertilized (20:20:20 N:P:K) every other week until bolting, at which point they were fertilized weekly. Survival until flowering and day of first flower was recorded.

Reproductive traits were scored on flowering individuals. Flower counts were carried out 14 days after first flower. Within a few days after first flower, mature anthers were removed prior to dehiscence from a single bud on each plant. Anthers were air‐dried and then placed in lactophenol‐aniline blue for at least 24 hrs in order to score pollen viability (Kearns & Inouye, 1993). Pollen viability was determined as the fraction of pollen grains stained, with an average of 595 ± 210(*SD*) grains scored per individual. Forty individuals had either <25 total grains of pollen or <3% viable pollen and were deemed male sterile. These individuals were not included in analyses of pollen viability, as moderate reductions in pollen viability and male sterility appeared to be separate, partially independent manifestations of reproductive isolation. All individuals that survived to flower were randomly crossed to two other individuals within that cross‐type, with each individual serving once as a pollen recipient and once as a pollen donor. Crosses within maternal families were avoided to minimize inbreeding. Most pollinations were carried out within 3 weeks of first flowering.

For each of the 24 between‐population crosses (eight within‐ and 16 between‐clade), hybrid performance relative to that of the parental populations was calculated for germination, survival to flower, flower number, seed number, pollen viability, and cumulative fitness. Cumulative fitness was calculated as (germination × survival × total flower # ×  (seed #+pollen viability)/2), with seed number standardized by dividing all seed counts by the largest seed count over all cross‐types.

### 2.3 Calculation of genetic distance

Genetic distance was calculated for both chloroplast and nuclear loci using data from a previous study on the phylogeography of *C. americanum* (Barnard‐Kubow et al., 2015). Chloroplast‐genetic distance for each cross was calculated as the number of SNPs between previously published parental haplotypes obtained via sequencing five chloroplast markers (Barnard‐Kubow et al., 2015). Nuclear‐genetic distance was calculated from previously published RAD‐seq data (Barnard‐Kubow et al., 2015). In brief, for all nine populations, DNA from six individuals was pooled, barcoded, and used for RAD‐seq library construction and sequencing. The program Stacks (Catchen, Amores, Hohenlohe, Cresko, & Postlethwait, 2011; Catchen, Hohenlohe, Bassham, Amores, & Cresko, 2013) was then used to identify and genotype loci. Only loci that were fixed within populations and variable between were used for subsequent analysis. For more details on RAD sequencing and genotyping, see Barnard‐Kubow et al. (2015). Using the fasta output from Stacks, maximum likelihood pairwise distances were calculated between all populations using RAxML (Stamatakis, 2014). These distances were used as the nuclear‐genetic distance for each of the crosses carried out in this study.

### 2.4 Statistical analysis

Patterns of hybrid performance and RI were first examined at the level of the individual cross. ANOVA was carried out for each trait to test for variation among the four cross‐types (two parental and two reciprocal hybrids) (PROC GLM; SAS 9.3 SAS Institute, INC., 2011). For pollen viability, log‐linear analyses were conducted assuming a gamma distribution and a log link (PROC GENMOD; SAS 9.3 SAS Institute, INC., 2011). If there was significant variation among cross‐types, linear contrasts were used to test for RI (whether hybrid performance was significantly reduced relative to the parents) and hybrid asymmetry (whether performance differed between hybrid reciprocals) within each cross. In cases of asymmetry, additional linear contrasts were run to test whether only one or both of the reciprocals had significant RI (i.e., performance of each reciprocal was significantly reduced relative to the parents). In these analyses, family means (*N* = 10–15 for most crosses) served as the data points. Similar family level ANOVA analyses were conducted in the four crosses that exhibited male sterility to evaluate reproductive isolation for male fertility. For calculating family means for cumulative fitness, families where all individuals died before being able to mature seed were assigned a seed count of zero. Similarly, families where all individuals were pollen sterile were assigned a pollen viability of zero.

Next, patterns of RI were examined by comparing the relative hybrid performance of each cross. Mean relative hybrid performance was determined for each cross by subtracting the mean of the two parental populations (midparent, *N* = 50 for most means) from the mean F1 hybrid value (*N* = 50 for most means) for each trait and then dividing that value by the midparent value to get a standardized percent hybrid deviation from the midparent value [((hybrid‐midparent)/midparent)  × 100]. This standardization allows for comparison among crosses. Negative values represent RI, while positive values represent heterosis or hybrid vigor. Similar calculations were also carried out for each F1 hybrid reciprocal separately (*N* = 25 for most means) to determine whether hybrid performance depended upon crossing direction.

The effect of crossing within versus between clades, and geographic, chloroplast‐, and nuclear‐genetic distance on variation in hybrid performance for each trait was determined using ANCOVA (*N* = 24, 8 within‐clade + 16 between‐clade crosses). Specifically, we tested whether crossing between genetic clades leads to greater RI (reduced hybrid performance) than crossing within clades. We also tested whether geographic, chloroplast‐genetic, or nuclear‐genetic distance between parental populations contributed to the strength of RI by including them in the model as direct effects. None of the tests for the interactions between genetic divergence (cross‐type) and the three measures of distance (covariate) were significant; therefore, the interaction terms were dropped and the multiple regression analyses rerun with only the four main effects. Analyses were conducted on mean relative hybrid performance for each cross for the six traits (germination, survival, flower number, seed number, pollen viability, and cumulative fitness) (PROC GLM; SAS 9.3 SAS Institute, INC., 2011). No significant correlations were detected among the measures of distance used in the ANCOVA analysis (geographic, chloroplast‐genetic, or nuclear‐genetic; PROC CORR; SAS 9.3, SAS Institute, INC., 2011).

To determine whether crossing direction influenced the magnitude of RI and/or the relationship of RI with geographic, chloroplast‐, or nuclear‐genetic distance, a similar ANCOVA analysis was run using just the between‐clade crosses (*N* = 32, 16 between‐clade crosses × 2 crossing directions). Separate measures of relative hybrid performance calculated for each F1 hybrid reciprocal were used and the analyses tested for an effect of crossing direction, the three measures of distance, included as direct effects, and the interactions between crossing direction and the measures of distance. The interaction effects were retained for these analyses as tests of the interactions between crossing direction and the three measures of distance (covariate) were highly significant (*p* < .01 and *p* < .001) for two of 18 analyses. Crossing direction was defined by whether parental populations belonging to the Appalachian clade were maternal or paternal (or the Eastern clade for crosses s86xe71, s90xe85, s88xe71, and s88xe85).

Finally, we compared the results from this study (crosses between Mountain lineages), to a previous range‐wide crossing study (Barnard‐Kubow et al., 2016) to examine patterns of RI across the full genetic and geographic range of *C. americanum*. While the Mountain and range‐wide studies covered similar levels of nuclear‐genetic (0.0010–0.0021 and 0.0007–0.0024, respectively) and chloroplast‐genetic (0–25 SNPs and 0–27 SNPs, respectively) divergence and included three overlapping populations, the Mountain study was restricted to a narrower geographic range (39–566 km vs. 43–1400 km).

## 3 RESULTS

**Figure 2:**
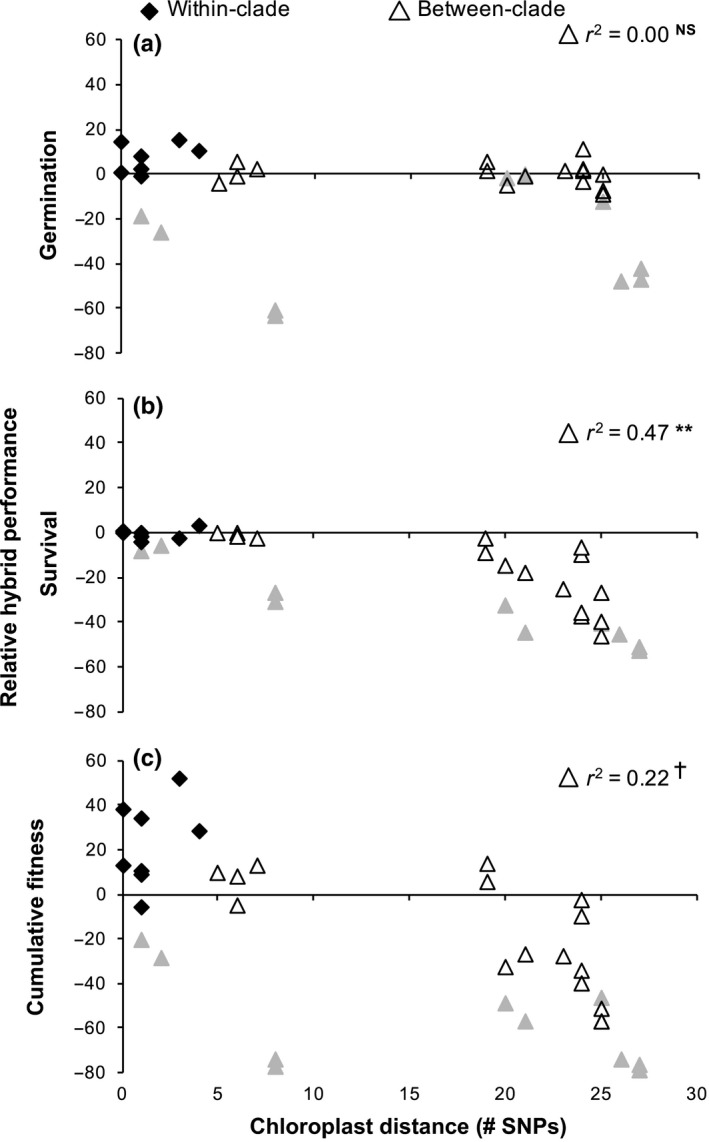
Performance of *C. americanum* hybrids relative to parental populations calculated as the percent reduction in hybrid values relative to the parents, such that negative numbers represent RI. Mean hybrid germination (a), survival (b), and cumulative fitness (c) for each cross graphed against chloroplast‐genetic distance. r^2^ values obtained from regression analysis of relative between‐clade hybrid values against chloroplast‐genetic distance. Smaller gray triangles depict relative hybrid performance for between‐clade crosses from the earlier range‐wide study.^†^ *p* < .07; **p < .01

**Figure 3:**
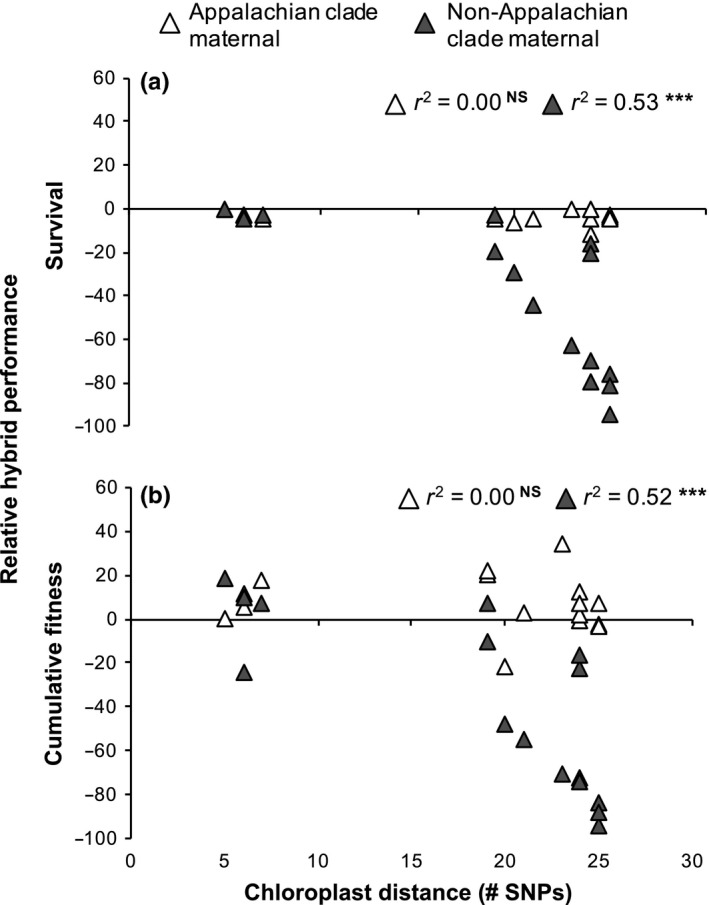
Survival (a) and cumulative fitness (b) of between‐clade *C. americanum* hybrids relative to parental populations for each crossing direction graphed against chloroplast‐genetic distance. Negative values represent RI. r^2^ values obtained from regression analysis of survival and cumulative fitness for each crossing direction against chloroplast‐genetic distance

Substantial postzygotic RI was found in between‐clade crosses (Figures 2 and 3), with up to a 94% reduction in cumulative fitness (Figure 3b). RI for cumulative fitness was primarily driven by reduced hybrid survival, the only individual fitness trait to show substantial RI (also up to 94% reduction) (Figure 3a). Chloroplast‐genetic distance between parental populations predicted strength of RI for survival (*r* ^2^ = .47, Table 1, Figure 2b), with larger distances generally leading to greater reductions in survival. Correspondingly, RI for cumulative fitness was also predicted by chloroplast‐genetic distance (*r* ^2^ = .22, Table 1, Figure 2c), although not as strongly, with larger distances again leading to a greater reduction in fitness. Geographic and nuclear‐genetic distance did not predict RI for any trait (Table 1). RI for survival and cumulative fitness in between‐clade crosses was strongly asymmetrical, with the occurrence of RI dependent upon crossing direction (Table 2, Figure 3). For all 10 crosses that showed RI for survival, a reduction in fitness was only observed when Smoky or Eastern populations were maternal (Table 3, Figure 3). Fitness reductions in this crossing direction were substantial (up to 94% reduction in survival). No reduction in survival was found when Appalachian populations were maternal.

**Table 1:** Testing for an effect of genetic divergence (crossing within or between clades), and magnitude of geographic, chloroplast‐genetic, or nuclear‐genetic distance between parental populations of *C. americanum*, on hybrid performance for fitness components. *F*‐values are reported with values significant at *p *<* *.05 shown in bold

Trait	Genetic divergence	Geographic distance	Chloroplast distance	Nuclear distance
Germination	1.87	0.13	0.21	0.01
Survival	0.23	0.58	**20.94** ***	2.80
Flower number	0.01	0.40	3.27^a^	0.01
Seed number	0.77	0.18	0.94	2.23
Pollen viability	2.42	2.12	3.91^a^	0.95
Cumulative fitness	1.10	2.74	**10.11** **	0.81

^a^ *p *< .1; *p <* *.05; ** *p *<* *.01; *** *p *<* *.001.

*df* 1,19.

**Table 2:** Comparison of crossing direction and magnitude of geographic, chloroplast‐genetic, or nuclear‐genetic distance between parental populations of *C. americanum*, on the strength of RI for fitness components of between‐clade crosses. *F*‐values are given for testing for an effect of crossing direction (which population is maternal), distance, as well as the interaction between the two. Values in bold are significant at *p *<* *.05

Trait	Direction	GeoDist	Direction*GeoDist	CpDist	Direction*CpDist	NucDist	Direction*NucDist
Germination	0.02	0.57	0.17	0.00	0.50	0.010	0.03
Survival	0.72	0.53	0.13	**15.04** ***	**14.44** ***	1.68	2.12
Flower number	0.19	0.81	0.60	2.36	1.17	0.12	0.29
Seed number	0.25	0.07	1.18	1.99	0.20	1.24	0.68
Pollen viability	0.03	2.30	0.11	**4.52** *	1.62	1.61	0.33
Cumulative fitness	0.12	1.42	0.38	**9.54** **	**9.13** **	0.16	0.25

*p <* *.05; **p <* *.01; ***p <* *.001.

*df* 1,24.

**Table 3:** Individual cross‐level analyses of hybrid performance of *C. americanum*, including the metric of cumulative fitness. Down arrows indicate crosses where hybrids performed significantly worse than their midparent (RI), while up arrows indicate significantly better performance (heterosis). In crosses where the reciprocal F1 hybrids were significantly asymmetrical, results for the reciprocals are shown separately, with the crossing direction where the Smoky or Eastern populations (first population listed under clade/cross) were maternal represented by arrows on the left, and the other crossing direction by arrows to the right. If no significant asymmetry was detected, then only one arrow is shown representing the results for the pooled hybrid population. Within clade, crosses are organized by geographic distance between populations with smaller distances at the top

Cross‐type	Clade	Germination	Survival	Flower #	Seed #	Pollen viability	Male fertility^a^	Cumulative fitness	Cross
Within	App			↑*					91x92
Within	App								5x73
Within	App	↑*		↑*				↑*	92x73
Within	App								5x91
Within	East		↓*					↑	85x71
Within	Smoky								88x90
Within	Smoky			↑*		↓*			90x86
Within	Smoky					↓*			88x86
Between	Smoky x East			↑*		↓*	↓*	↓*(↑	86x71
Between	Smoky x East						↓*	↓	90x85
Between	Smoky x East								88x85
Between	Smoky x East								88x71
Between	East x App		↓*	↓*				↓*	85x5
Between	East x App		↓*	↓*				↓*	71x73
Between	East x App								85x91
Between	East x App		↓*						71x92
Between	Smoky x App		↓*	↓*	↓			↓*	88x91
Between	Smoky x App		↓*						90x91
Between	Smoky x App		↓*	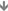				↓*	88x92
Between	Smoky x App	↑*	↓*			↓*	↓*	↓*	86x73
Between	Smoky x App		↓*	↓↑*	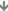	↓*		↓*)↑	86x92
Between	Smoky x App	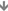	↓*	↓*				↓*	88x73
Between	Smoky x App		↓*					↓*	88x5
Between	Smoky x App						↓*	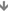	90x5

Gray: *p *<* *.075, black *p *<* *.05, *p <* *.0125 (significant after correction for multiple nonindependent contrasts).

^a^Male sterility was not included in cumulative fitness.

Moderate RI for pollen viability was also found in a subset of crosses (10–15% reduction in four crosses, 34% reduction in one cross; Table 3; Fig. S1). In contrast to survival, reduced pollen viability occurred in both within‐ and between‐clade crosses (Table 3). In addition, all five crosses that exhibited reduced pollen viability involved a single Smoky‐clade population, s86 (Table 3). While chloroplast‐genetic distance significantly affected pollen viability when examining the F1 hybrid reciprocals separately (Table 2), it appeared as though one cross, s86xe71, with a 34% reduction, was driving this effect (Fig. S1). Further analysis found that this cross was acting as a strong outlier in the multiple regression analysis (Cook's *D* = 1.0) and removing the cross from the analysis eliminated the effect of chloroplast‐genetic distance on RI for pollen viability. Therefore, reduced pollen viability was not well predicted by any measure of genetic or geographic distance.

**Figure 4:**
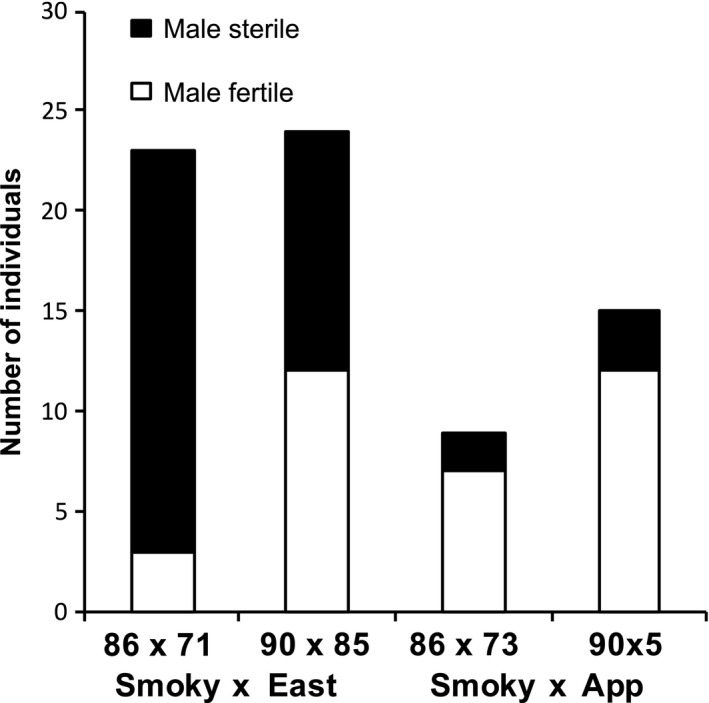
Patterns of male sterility in four *C. americanum* between‐clade crosses. For each cross, the number of hybrid individuals is shown that were male sterile (no viable pollen) or male fertile (viable pollen). Data are only shown for the direction of the cross where the Smoky populations were maternal, as male sterility did not occur in the other crossing direction. Population IDs are listed for each cross, as well as the type of between‐clade cross to which they belong

Male sterility also occurred in four between‐clade crosses (Table 3; Figure 4), partially independent of the RI for pollen viability. Each of these four crosses involved a Smoky‐clade population with chloroplast haplotype I (Table S1) and either an Eastern or Appalachian clade population. The occurrence of male sterility was also strongly asymmetrical, with male sterile individuals only occurring when the Smoky‐clade populations were maternal. The frequency of pollen sterile individuals ranging from 20 to 87% (Figure 4). Two of the crosses containing pollen sterile individuals (s86xe71 and s86xe73, Figure 4) also exhibited significant RI for pollen viability (Table 3).

## 4 DISCUSSION

Substantial reproductive isolation was found when crossing between the three Mountain lineages of *C. americanum*, with up to a 94% reduction in cumulative fitness. Reproductive isolation was primarily due to reduced survival, although moderate reductions in pollen viability and male fertility were also found in a subset of crosses. These findings indicate that multiple genetic incompatibilities exist between *C. americanum*'s divergent Mountain lineages even though the lineages co‐occur within a relatively narrow geographic range. Combining these results with those of an earlier range‐wide study (Barnard‐Kubow et al., 2016) demonstrates the existence of four distinct genetic incompatibilities that influence reproductive isolation across the life cycle in *C. americanum*. Each incompatibility exhibits a different pattern of occurrence and expression across the species range, giving insight into whether these incompatibilities are cytonuclear or nuclear‐nuclear in origin and suggesting they are evolving due to a variety of intrinsic genetic factors and extrinsic environmental factors.

### 4.1 Cytonuclear driver of reduced survival

**Figure 5:**
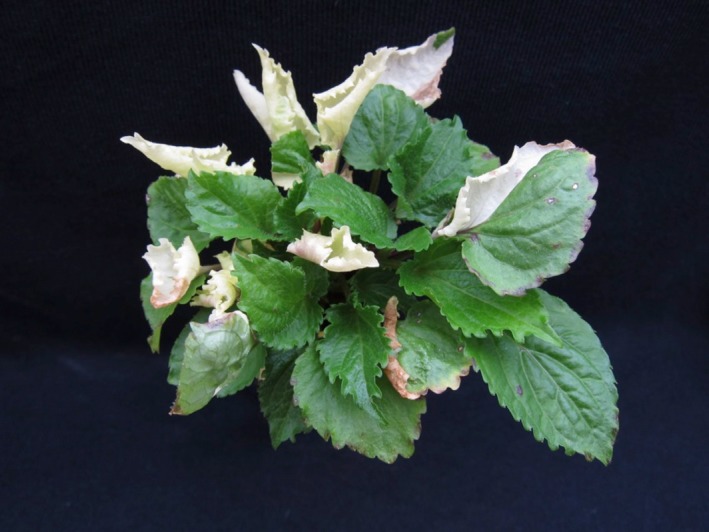
Example of a hybrid variegated *C. americanum* rosette resulting from a cross between genetic lineages

Reproductive isolation among the Mountain lineages was almost entirely driven by reductions in survival (up to 94% reduction). Strength of reproductive isolation for survival was predicted by chloroplast‐genetic distance and was highly asymmetrical, indicating a role for the cytoplasm in this genetic incompatibility. Hybrids were frequently chlorotic (lacking chlorophyll) or variegated (Figure 5), further supporting cytonuclear incompatibility, as variegation occurs in species with biparental chloroplast inheritance (such as *C. americanum*) when one of the two chloroplasts is incompatible on the hybrid nuclear background (Bogdanova, 2007; Greiner et al., 2011; Kirk & Tilney‐Bassett, 1978; Ureshino et al., 1999; Weihe, Apitz, Pohlheim, Salinas‐Hartwig, & Borner, 2009). Accordingly, reduced survival in Mountain lineage hybrids appears to be caused by cytonuclear incompatibility, where the Smoky/Eastern chloroplasts are incompatible on the hybrid nuclear background. Although postzygotic reproductive isolation is often strongest for fertility traits (Brys, Broeck, Mergeay, & Jacquemyn, 2014; Lowry et al., 2008; Martin & Willis, 2010; Sambatti, Strasburg, Ortiz‐Barrientos, Baack, & Rieseberg, 2012; Scopece, Widmer, & Cozzolino, 2008), cytonuclear incompatibilities frequently influence viability and are often expressed in the F1 generation (Burton et al., 2013; Greiner et al., 2011; Levin, 2003; Turelli & Moyle, 2007).

Substantial reductions in survival due to cytonuclear incompatibility were also found between Western and Mountain lineages in the range‐wide study (Barnard‐Kubow et al., 2016). A consistent relationship between reduced survival and chloroplast‐genetic distance with no influence of geographic distance indicates that time since divergence is the primary factor influencing incompatibility, as opposed to extrinsic factors such as local adaptation to differing environments. One intrinsic process driving the evolution of cytonuclear incompatibility in *C. americanum* could be its rapid rate of plastid genome evolution (Barnard‐Kubow, Sloan, & Galloway, 2014; Barnard‐Kubow et al., 2016). Rapid organelle genome evolution has the potential to facilitate the evolution of cytonuclear incompatibility by leading to accelerated co‐evolution of organelle and nuclear genomes within populations, and thus incompatibility between populations (Barnard‐Kubow et al., 2014, 2016; Burton et al., 2013; Greiner et al., 2011; Hill, 2016; Levin, 2003).

While the overall pattern of reproductive isolation for survival was consistent across the two studies, there were also striking differences. The Western and Smoky lineages group together in *C. americanum*'s chloroplast phylogeny. As reduced survival is driven by cytonuclear incompatibility, one would predict that Western and Smoky populations would lead to similar reductions in survival when crossed with Eastern and Appalachian populations. However, reduced survival was only observed when crossing Eastern populations to Western populations (27–31% reduction) but not Smoky populations (0–2% reduction). This difference was more pronounced for cumulative fitness which was reduced by 74–78% in the two Western by Eastern clade crosses, but ranged from a 5% reduction to a 12% increase in the four Smoky by Eastern clade crosses. Finally, crossing Western populations to Appalachian populations consistently led to reduced survival (40–53% reduction), while crossing Smoky to Appalachian populations resulted in more variable reductions (6–46% reduction).

What might underlie these differences between the Western and Smoky lineages? While the Smoky populations do group with the Western populations in the chloroplast phylogeny, they group with the Eastern populations in the nuclear phylogeny. The Smoky populations are also geographically distinct from the Western lineage, co‐occurring with the other Mountain clades. Finally, Smoky populations are found within 5 km from populations of other Mountain lineages (C. Debban, personal communication). One possibility is that the Smoky populations have a Western origin, but have since experienced secondary contact and gene flow with Eastern and/or Appalachian lineage populations, leading to the differing topologies of the nuclear and chloroplast trees. Accordingly, Smoky populations may have been incompatible with Eastern populations at some point in the past, but introgression has since reduced this incompatibility. Variable introgression could also explain why Smoky populations are not universally incompatible with Appalachian populations. Alternatively, the Smoky populations may have diverged from the Western populations early during re‐colonization, before the incompatibility between the Western and Eastern clades evolved. The Smoky populations contain chloroplast haplotypes that are basal in the Western lineage (Barnard‐Kubow et al., 2015), suggesting a potential early divergence. Future studies could test these alternative hypotheses.

### 4.2 Reproductive isolation for pollen viability unrelated to geographic or genetic distance

Among the Mountain lineages moderate, symmetrical reductions in pollen viability were observed in four crosses. This reproductive isolation occurred in both within‐ and between‐clade crosses and was unrelated to geographic or genetic distance, consistent with that found in the range‐wide study (Barnard‐Kubow et al., 2016). This consistent lack of relationship with chloroplast‐genetic or geographic distance, as well as the symmetry of the reproductive isolation suggests a nuclear incompatibility driven primarily by intrinsic factors. Similar moderate, symmetrical reductions in pollen viability that are polymorphic among populations are found in *Mimulus* and interpreted to indicate nuclear Bateson‐Dobzhansky‐Muller incompatibilities that are selectively neutral or of relatively recent origin (Martin & Willis, 2010). The stochastic appearance of reproductive isolation for pollen viability between populations of *C. americanum* suggests the underlying alleles may similarly be selectively neutral or of relatively recent origin.

### 4.3 Geographically restricted male sterility as another example of cytonuclear incompatibility

Reproductive isolation for male fertility was found among the Mountain lineages in a narrow geographic part of the range. Male sterility was only found in crosses involving two Smoky populations with the same chloroplast haplotype (I) when that population was maternal, suggesting a cytoplasmic contribution and therefore cytoplasmic male sterility (CMS; Chase, 2007). While CMS has not previously been documented in *C. americanum*, studies in other taxa have found that crosses between closely related species can reveal CMS in hermaphrodites (Fishman & Willis, 2006; Leppala & Savolainen, 2011; Levy, 1991). No evidence for CMS was found in the earlier, range‐wide crossing study.

Finding CMS in intraspecific crosses of an otherwise hermaphroditic species may be the signature of past cytonuclear conflict, where both a sterilizing cytoplasm and a corresponding nuclear restorer have since gone to fixation within populations (Case & Willis, 2008; Fishman & Willis, 2006). Interestingly, CMS in *C. americanum* occurred when crossing the haplotype I Smoky populations to the Eastern and the northern Appalachian populations, but not when crossing to the southern Appalachian populations. This difference likely reflects genetic divergence between northern and southern Appalachian populations (Barnard‐Kubow et al., 2015). In addition, pollen sterility varied among hybrids from 20 to 87%, suggesting the existence of genetic variation within and among populations for either the mitochondrial CMS‐determining alleles or the nuclear restorers (Leppala & Savolainen, 2011; Sweigart, Mason, & Willis, 2007). One possibility is that within these Smoky populations, both the sterilizing cytoplasm and the corresponding nuclear restorer have gone to fixation, but the nuclear restorer is variable within the Eastern and Appalachian populations to which they were crossed. Gene flow through pollen generally occurs over greater geographic distances than gene flow through seed (Petit et al., 2005; Pinheiro et al., 2013), which could explain why the restorer, but not the sterilizing cytoplasm, may be found in these more distant Eastern and Appalachian populations.

### 4.4 Reproductive isolation for germination may be driven by extrinsic factors

No reproductive isolation for germination was found when crossing among the Mountain lineages. In contrast, range‐wide RI for germination increased with the geographic distance among parental populations (Barnard‐Kubow et al., 2016), suggesting local adaptation to differing environments may have contributed to the evolution of the incompatibility. However, all of the crosses that exhibited reduced germination in the range‐wide study also involved a Western population, leading to a confounding of geographic distance and the influence of the Western lineage. The current study involved no Western populations. While the crosses reported here predominately occurred across shorter geographic distances than the range‐wide study, five were across distances above the threshold for reproductive isolation in the earlier study (400–600 km), and yet exhibited no signs of reduced germination. Specifically, these five exhibited only a 0–1% reduction in germination, while a 12–42% reduction was observed over the same geographic distances in the range‐wide study. This suggests that reproductive isolation for germination may be due to a feature specific to the Western lineage rather than geographic separation.

The distribution of the Western clade is geographically and environmentally distinct from that of the Mountain clades and covers a wider range of environments. Therefore, the genetic incompatibility underlying reduced germination could be a by‐product of adaptation of the Western clade to this broader range of environments. In addition, the Western clade experienced larger‐scale migration after the most recent glacial maximum than the Mountain clades (Barnard‐Kubow et al., 2015). Sequential bottlenecks due to re‐colonization have the potential to reduce genetic diversity and increase genetic drift, potentially contributing to the evolution of genetic incompatibility. Altogether, it appears that in the case of *C. americanum,* extrinsic factors influencing adaptation, migration, and demography are the likely cause of the genetic incompatibility underlying reduced germination. This finding is somewhat in contrast to recent studies, where disrupted parental imprinting and endosperm development have frequently been found to underlie seed inviability (Garner, Kenney, Fishman, & Sweigart, 2016; Josefsson, Dilkes, & Comai, 2006; Rebernig, Lafon‐Placette, Hatorangan, Slotte, & Köhler, 2015).

### 4.5 Consequences for speciation

By combining the results from two crossing studies and characterizing reproductive isolation across the full geographic and genetic range of *C. americanum*, we have demonstrated the complexity of the initial stages of speciation. Within *C. americanum* there are multiple, independent genetic incompatibilities leading to reproductive isolation in a range of traits, expressed across the entire life cycle, from germination to reproduction. It is also worth noting that two of these four incompatibilities are cytonuclear incompatibilities, indicating an important role for this type of incompatibility early in speciation. The presence of multiple, independent incompatibilities evolving simultaneously in different geographic areas of the species range indicates, perhaps unsurprisingly, that the view of speciation as a simple bifurcation is likely overly simplistic. Instead, early speciation may be more accurately characterized as a mosaic of genetic divergence and reproductive isolation across a species range.

This mosaic likely has implications for the outcome of speciation, as even though Mountain lineages are as reproductively isolated from one another as from the Western lineage in terms of cumulative fitness (up to 94% reduction in both studies), the number of incompatibilities underlying this reproductive isolation differs. Mountain lineage hybrids consistently exhibit reproductive isolation only in terms of reduced survival, whereas Mountain by Western hybrids consistently exhibits reproductive isolation for both germination and survival. Thus, even though these lineages appear to be equally reproductively isolated in terms of cumulative fitness, if Mountain and Western lineages come into secondary contact they may be more likely to maintain genetic divergence than if there is secondary contact between Mountain lineages, as Mountain by Western hybrids must overcome multiple barriers to gene flow, while Mountain lineage hybrids must only overcome a single barrier. Further, incompatibilities with a more stochastic pattern, such as pollen viability and male sterility, will add an additional idiosyncratic element to reproductive isolation creating stronger barriers between some populations, further contributing to the shifting mosaic of reproductive isolation and outcome of speciation.

## CONFLICT OF INTEREST

None declared.

## AUTHOR CONTRIBUTIONS

KBB and LFG conceived of and designed the study. KBB carried out the study. KBB and LFG both carried data analysis and drafted the manuscript.

## Supporting information


** **

** **
