# Supplementary figures and images for: Variation in reproductive isolation across a species range
Source: Ecol Evol. 2017 Oct 7;7(22):9347–57. doi: 10.1002/ece3.3400 (PMC5696433; doi:10.1002/ece3.3400)

**Relative Hybrid Performance  
For Pollen Viability**

◆ Within-clade

△ Between-clade

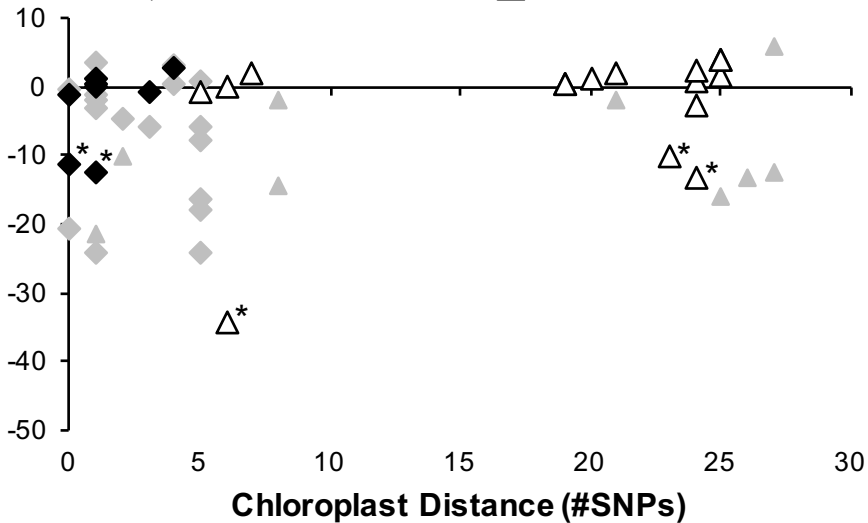

Supplement: Supplementary file 1 [file ECE3-7-9347-s001.pdf]
