# Supplementary material for: Variation in reproductive isolation across a species range
Source: Ecol Evol. 2017 Oct 7;7(22):9347–57. doi: 10.1002/ece3.3400 (PMC5696433; doi:10.1002/ece3.3400)
Supplement: Supplementary file 2 [file ECE3-7-9347-s002.docx]

**Supplementary Table 1:** Information for the *C. americanum* populations in the eastern United States used in the experiment to evaluate reproductive isolation including phylogenetic clade, geographic location and chloroplast haplotype (Barnard-Kubow *et al.*, 2015).

| **Clade** | **Pop ID** | **State** | **Latitude** | **Longitude** | **Haplotype** |
| --- | --- | --- | --- | --- | --- |
| Appalachian | 91 | North Carolina | 35.5862 | -83.0663 | A |
| Appalachian | 92 | Tennessee | 35.6758 | -83.5259 | B |
| Appalachian | 5 | Maryland | 39.6137 | -79.1158 | D |
| Appalachian | 73 | Virginia | 37.3534 | -80.5522 | D |
| East | 71 | Virginia | 38.3305 | -78.4901 | E/F |
| East | 85 | Virginia | 37.7576 | -79.1876 | E |
| Smoky | 90 | North Carolina | 35.7669 | -82.1636 | I |
| Smoky | 86 | Virginia | 36.6344 | -81.5881 | I |
| Smoky | 88 | Tennessee | 35.9833 | -82.4989 | J |
